# Increased Risk for *Entamoeba histolytica* Infection and Invasive Amebiasis in HIV Seropositive Men Who Have Sex with Men in Taiwan

**DOI:** 10.1371/journal.pntd.0000175

**Published:** 2008-02-27

**Authors:** Chien-Ching Hung, Dar-Der Ji, Hsin-Yun Sun, Ya-Tien Lee, Shui-Yuan Hsu, Sui-Yuan Chang, Cheng-Hsin Wu, Yun-Hsien Chan, Chin-Fu Hsiao, Wen-Chun Liu, Robert Colebunders

**Affiliations:** 1 Department of Internal Medicine, National Taiwan University Hospital and National Taiwan University College of Medicine, Taipei, Taiwan; 2 Research and Diagnostic Center, Centers for Disease Control, Department of Health, Taipei, Taiwan; 3 Department of Clinical Laboratory Sciences and Medical Biotechnology, National Taiwan University College of Medicine, Taipei, Taiwan; 4 Division of Biostatistics and Bioinformatics, National Health Research Institutes, Zhunan Town, Taiwan; 5 Institute of Tropical Medicine, Antwerp, Belgium; 6 University of Antwerp, Antwerp, Belgium; Jawaharlal Nehru University, India

## Abstract

**Background:**

Incidence of *Entamoeba histolytica* infection and clinical manifestations and treatment response of invasive amebiasis (IA) in HIV-infected patients have rarely been investigated before.

**Methodology/Principal Findings:**

At the National Taiwan University Hospital, medical records of HIV-infected patients who received a diagnosis of IA between 1994 and 2005 were reviewed. The incidence of amebiasis was investigated in serial blood and stool samples from 670 and 264 HIV-infected patients, respectively, using serological and specific amebic antigen assays. DNA extracted from stool samples containing *E. histolytica* were analyzed by PCR, sequenced, and compared. Sixty-four (5.8%) of 1,109 HIV-infected patients had 67 episodes of IA, and 89.1% of them were men having sex with men (MSM). The CD4 count at diagnosis of IA was significantly higher than that of the whole cohort (215 cells/µL vs. 96 cells/µL). Forty episodes (59.7%) were liver abscesses, 52 (77.6%) colitis, and 25 (37.3%) both liver abscesses and colitis. Fever resolved after 3.5 days of metronidazole therapy (range, 1–11 days). None of the patients died. The incidence of *E. histolytica* infection in MSM was higher than that in other risk groups assessed by serological assays (1.99 per 100 person-years [PY] vs. 0 per 100 PY; *p*<0.0001) and amebic antigen assays (3.16 per 100 PY vs. 0.68 per 100 PY; *p* = 0.12). In multiple logistic regression analysis, only MSM was significantly associated with acquisition of *E. histolytica* infection (adjusted odds ratio, 14.809; *p* = 0.01). Clustering of *E. histolytica* isolates by sequencing analyses from geographically-unrelated patients suggested person-to-person transmission.

**Conclusions/Significance:**

HIV-infected MSM were at significantly higher risk of amebiasis than patients from other risk groups. Despite immunosuppression, amebic liver abscesses and colitis responded favorably to treatment.

## Author Summary

*Entamoeba histolytica*, morphologically identical to but genetically different from *E. dispar* and *E. moshkovskii*, is the causative agent of amebiasis. Recently there have been reports of increased risk for amebiasis among men who have sex with men (MSM) due to oral-anal sexual contact in several developed countries. In this longitudinal follow-up study, the incidence of amebiasis was determined among HIV-infected patients using serological and specific amebic antigen assays. DNA extracted from stool samples containing *E. histolytica* were analyzed by PCR, sequenced, and compared. Clinical manifestations and treatment response of invasive amebiasis in HIV-infected patients were reviewed. The results demonstrated that HIV-infected MSM were at significantly higher risk of amebiasis than patients from other risk groups. Clustering of *E. histolytica* isolates by sequencing analyses from geographically unrelated patients suggested person-to-person transmission. Despite immunosuppression, amebic liver abscesses and colitis responded favorably to metronidazole therapy. It is important to investigate in areas of high incidence of both amebiasis and HIV (sub-Saharan Africa) how generalizable these findings are.

## Introduction

Invasive amebiasis (IA) is the second most common cause of mortality due to parasite infections worldwide, accounting for 40,000 to 100,000 deaths annually. High risk populations for developing IA include infants, pregnant women, and patients who are taking immunosuppressives [1],[2]. Interestingly, IA has not been considered to occur at a higher frequency in HIV-infected patients [3],[4]. In industrialized countries, the rare occurrence of IA in HIV-infected patients or persons at risk for HIV infection is probably attributed to the rare intestinal carriage of *E. histolytica* [4]–[9]. This is in contrast with the relatively frequent carriage of the non-pathogenic *E. dispar* among men who have sex with men (MSM) who attend sexually transmitted diseases clinics [10]–[13]. In a retrospective review of medical records of more than 34,000 HIV-infected patients in the US [9], 111 (0.3%) patients were diagnosed as having *E. histoytica* or *E. dispar* infection, and only 2 had extra-intestinal amebiasis. Amebiasis was significantly more prevalent among MSM and patients from *E. histolytica* endemic areas. However, the interpretation of the results of this study is limited by the retrospective study design and failure to differentiate between *E. histolytica* and *E. dispar* [14].

In developing countries, studies comparing the prevalence of amebiasis in HIV-infected and HIV-uninfected persons yielded inconsistent results [15]–[22]. The interpretation of these studies, however, is difficult because a majority of the diagnosis of amebiasis was based solely on microscopic examination of stool samples, which is an insensitive test that fails to distinguish *E. histolytica* from *E. dispar* [14]. In a cross-sectional study using stool antigen detection and polymerase chain reaction (PCR) from Mexico, where amebiasis is endemic, investigators found that HIV-infected patients appeared to have a higher rate, though not statistically significant, of *E. histolytica* infection than their sexual partners or close contacts [23]. However, those patients colonized with *E. histolytica* did not develop invasive diseases over the 12-month follow-up period.

Over the past few years, we and many investigators in Japan, Taiwan, and Korea have found that IA is increasingly diagnosed among HIV-infected MSM [24]–[31]. Of the estimated 500 to 600 reported cases of amebiasis annually in Japan, 80% of them occurred in MSM [32] and a substantial proportion of patients with IA were also co-infected with HIV and syphilis [24],[30]. In Taiwan, an estimated 5–6% of HIV-infected patients developed IA, and in many IA was the presenting disease of HIV infection [31]. Serologic surveys in the US, Italy, Japan, and Taiwan also demonstrated that MSM, regardless of HIV status, were at an increased risk of exposure to *E. histolytica* [13], [26], [31], [33]–[35]. Recent detection of locally acquired amebiasis among MSM who had no recent travel to endemic areas for *E. histolytica* has raised concerns in Sydney, Australia [36]. Oral-anal sexual contact has been found to be significantly associated with acquisition of *E. histolytica* infection [37]. Although IA has been considered an increasingly important parasitic infection in HIV-infected patients in three East Asian countries, the incidence of amebiasis and the clinical spectrum and the response of IA to standard metronidazole therapy have not been well studied.

In this study, we conducted a longitudinal follow-up study to assess the incidence of *E. histolytica* infection among persons with HIV infection at a referral medical center for HIV care in Taiwan. We also described the clinical spectrum and treatment outcome of IA.

## Methods

### Retrospective review of invasive amebiasis cases

Medical records of 1109 consecutive, non-hemophiliac HIV-infected patients aged 15 years or greater were reviewed to identify cases of IA at the National Taiwan University Hospital from June 1994 to December 2005 with the use of a standardized case record form. Of the 1109 patients, 781 (70.4%) were MSM. During the study period, a standardized protocol was followed to investigate HIV-infected patients who presented with gastrointestinal symptoms [28],[31]. Those investigations included at least two stool specimens for bacterial cultures and microscopy of concentrated wet mount preparations and modified acid-fast staining; indirect hemagglutination (IHA) assay to detect anti-*E. histolytica* antibodies (Cellognostics, Boehhringer Diagnostics GmbH, Marburg, Germany); endoscopy and biopsy for histopathologic examinations in patients whose stool examinations were non-diagnostic; abdominal sonography followed by computed tomography for patients with abnormal liver function tests, and space-occupying lesions of the liver. Specific *Entamoeba* antigen assays using commercial test kits (ENTAMOEBA TEST, TechLab, Branchburg, NJ) followed by polymerase chain reactions (PCR) using specific primers for *E. histolytica* was introduced after 1 January, 2001 [31].

Definite IA was diagnosed when erythrophagocytic trophozoites and/or positive PCR to *E. histolytica* were identified in clinical specimens from patients with symptoms compatible with IA, such as colitis and liver abscesses [28],[38]. Probable IA was confirmed when a patient with IA symptoms responded to metronidazole monotherapy and the aspirates or blood specimens showed high IHA titers, but microbiological cultures for bacteria, fungi, or histopathological examination of aspirates and biopsy specimens did not reveal any other pathogen. Results of the IHA assay were considered positive if the titer was 128 or greater. AIDS was defined according to the 1993 revised classification system for HIV infection and expanded surveillance case definition for AIDS among adolescents and adults [39]. Highly active anti-retroviral therapy (HAART) was defined as anti-retroviral therapy containing two nucleoside reverse transcriptase inhibitors and a (boosted) protease inhibitor(s) or a non-nucleoside reverse transcriptase inhibitor.

### Sero-incidence of *E. histolytica* infection

Serum samples from HIV-infected patients at baseline were tested for IHA and patients who remained in clinic follow-up from January 2001 to December 2005 were re-tested to determine the sero-incidence of *E. histolytica* infection. The interval between the two blood samples was at least one year. In patients with samples that tested positive at the last clinic follow-up or the end date of the study (31 December, 2006), serially stored serum samples were retrospectively tested to determine the seroconversion date. Seroconversion was defined as changes from sero-negative at baseline to IHA titers of 128 or greater at subsequent IHA assay; or increases of IHA titers by four-fold or greater. The seroconversion date was defined as the mid-point between the dates when the last sero-negative sample and the first sero-positive sample were collected.

### Incidence of intestinal infection with *E. histolytica*

Sequential stool samples from HIV-infected persons were tested for the presence of stool *Entamoeba* antigen between 1 January, 2001 and 31 December, 2005. Those patients who were negative for *Entamoeba* antigen were asked to provide stool samples for follow-up testing using the same method in order to assess the incidence of new acquisition of *E. histolytica.*The interval between the two stool samples were at least 6 months. The date of new infection was estimated as the mid-point between the date when the last antigen-negative sample and the first antigen-positive sample were collected.

Stool specimens tested positive for *E. histolytica*/*E. dispar* antigen were further confirmed by PCR. The primer sets for a multiplex nested PCR were based upon the variable regions between 16S-like rDNAs of *E. histolytica* (GenBank X56991) and *E. dispar* (GenBank Z49256) [31]. The procedures to isolate total DNA from the stool samples and the PCR conditions were described previously [31]. Individual *E. histolytica* isolates were genotyped by PCR amplification and sequencing of the previously described polymorphic loci, *locus* 1-2, using one set of primers (R1: CTGGTTAGTATCTTCGCCTGT and R2: CTTACACCCCCATTAACAAT) previously described [40],[41]. PCR was carried out in a 50 µl reaction mixture containing 0.1 µg of DNA, a 1.5 µM concentration of each primer, 2.5 mM MgCl_2_, a 100 µM concentration of each deoxynucleoside triphosphate, and 1.5 U of AmpliTaq Gold DNA Polymerase (Applied Biosystems) with the *Taq* activation at 95°C for 15 min and 30 cycles of denaturation at 94°C for 30 s, annealing at 45°C for 30 s, and extension at 72°C for 1 min, and then final extension at 72°C for 10 min [40]. The PCR products were fractionated by electrophoresis in 3% NuSieve 3∶1 agarose (Cambrex, East Rutherford, USA), stained by ethidium bromide and visualized under UV illumination. After purification using the QIAquick PCR purification kit (QIAGEN), *locus* 1-2 PCR products were sequenced twice in the forward and reverse directions. The sequences from representative genotypes chosen to infer the phylogenetic trees of *locus* 1-2 were were manually edited and aligned by using BioNumerics V. 4.01 software (Applied Maths, Kortrijk, Belgium). The study protocols were approved by the Institutional Review Board of NTUH and patients gave written informed consent.

### Statistical analysis

All statistical analyses were performed using SAS statistical software (Version 8.1, SAS Institute Inc., Cary, NC, U.S.A.). Categorical variables were compared using χ^2^ or Fisher's exact test and non-categorical variables were compared using Wilcoxon's rank-sum test. The incidence rate of *E. histolytica* infection or seroconversion was calculated as number of episode per 100 person-years (PY) of observation. Exact 95% confidence intervals (95% CI) for incidence rates were calculated on the basis of the Poisson distribution. The follow-up duration was from the date with the first stool or blood sample that was negative for *E. histolytcia* antigen or IHA to the date with the sample that was positive, date of death, or on 31 December, 2006, whichever occurred first. Multiple logistic regression analysis was performed between patients who were diagnosed with newly acquired *E. histolytica* infection by serologies or antigen assays and those who remained uninfected in order to identify the risk factors associated with *E. histolytica* infection. All tests were two-tailed. A *p* value <0.05 was considered significant.

## Results

### Cases of invasive amebiasis

**Figure 1:**
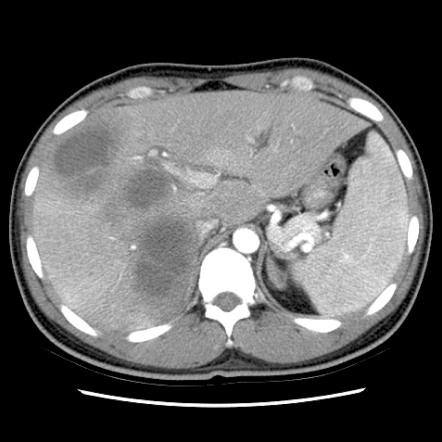
Abdominal computed tomography showing multiple liver abscesses of a 28-year-old male homosexual who presented with right upper quadrant pain, vomiting, and watery diarrhea for 4 days. Diagnosis of amebic liver abscess was confirmed by positive PCR for *Entamoeba histolytica* of the liver abscess aspirate. The titer of indirect hemagglutination antibody for *E. histolytica* was 8192.

**Figure 2:**
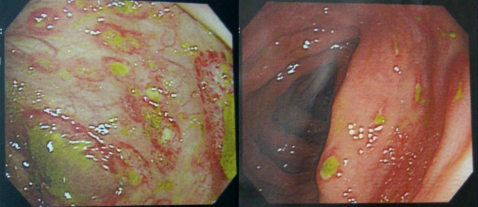
Colonoscopy of the same patient showing multiple ulcers at the cecum, and ascending, transverse, and descending colon.

During the 11-year study period, 64 (5.8%) HIV-infected patients were diagnosed as having 67 cases of IA (Table 1). All of the 64 patients were males and 57 (89.1%) were MSM. MSM had a higher risk of invasive amebiasis compared with other risk groups: 57/781 vs. 7/328 (risk ratio, 3.42; 95% CI, 1.5777, 7.417). In 29 cases (43.8%), HIV infection was concurrently diagnosed with IA. The CD4 count at diagnosis of IA was significantly higher than that of the whole cohort (215 cells?L vs. 96 cells?L). Fever (72.6%), diarrhea (70.8%), right upper quadrant pain (32.3%), and dysentery (20.6%) were the most common symptoms of IA. Fifty-two (77.6%) of the 67 IA episodes were amebic colitis, 40 (59.7%) episodes were liver abscesses (including 4 multiple abscesses), and 25 (37.3%) were both amebic liver abscesses and colitis (Figures 1 and 2). By IHA assays, 51.6% of the patients with IA had titers≧512 (range, 0–16384). Eight (11.9%) developed serious complications necessitating surgical intervention, which included 3 intestinal perforations and peritonitis, 2 ruptures of the liver abscess, 2 subphrenic abscesses, 1 empyema, and 1 hepatogastric fistula. Metronidazole was administered for 13 days (range, 3–27 days) and the interval from initiation of metronidazole to defervescence was 3.5 days (range, 1–11 days). Thirty-five patients received concurrent antibiotic therapy, mainly ceftriaxone, and the fever resolved after 2 days of antibiotic therapy (range, 0–10 days). Of 21 patients receiving only metronidazole, the fever resolved after 3 days of therapy (range, 1–6 days). Liver aspiration and drainage was performed in 14 (20.9%) patients. Two required a laparotomy and chest tube drainage. Iodoquinol was administered to 42 (62.7%) patients following completion of metronidazole therapy to clear intestinal colonization and prevent relapse. Nobody died of IA after a median observation of 748 days (range, 9–4179 days).

**Table 1:** Clinical characteristics of 64 patients with 67 episodes of invasive amebiasis.

Variable (No. of data available)	N (%) or Median (range)
Route of HIV transmission (67)	
Homosexual/bisexual	57 (85.1)
Heterosexual	10 (14.9)
Age at diagnosis (65)	34.0 (21–72)
CD4 at diagnosis (56)	198.0 (1–294)
PVL at diagnosis (log_10_ copies/ml)* (40)	5.0 (1.7–6.1)
HAART use at diagnosis* (65)	11 (16.9)
Clinical presentation	
Fever (63)	45 (71.4)
Diarrhea (65)	46 (70.8)
Watery stool (62)	31 (50)
Dysentery (63)	13 (20.6)
Abdominal pain (63)	39 (61.9)
Right upper quadrant pain (63)	20 (31.7)
Cough (63)	8 (12.7)
Laboratory	
White blood cell count (/l) (53)	8740.0 (2110–38140)
Hemoglobin (g/dL) (53)	11.3 (4.7–16.2)
GOT (IU/L) (52)	38.0 (11.0–1090.0)
GPT (IU/L) (47)	28.0 (5.0–856.0)
ALP (IU/L) (46)	190.5 (25.0–722.0)
Total bilirubin (mg/dL) (49)	0.51 (0.1–2.1)
Stool exam positive for *E. histolytica*/*E. dispar* (51)	10 (19.6)
Stool *E. histolytica*/*E. dispar* Ag (28)	7 (25.0)
Stool PCR positive for *E. histolytica* (27)	3 (11.1)
Colonoscopy showing colon ulcers (23)	6 (26.1)
Abdominal sonography (55)	
Right lobe abscess	26 (51.0)
Left lobe abscess	9 (17.6)
Disease involvement	
Liver abscess (40)	
Definite	5 (12.5)
Probable	35 (87.5)
Colitis (52)	
Definite	20 (38.5)
Probable	32 (61.5)
Treatment	
Metronidazole (61)	60 (98.4)
Metronidazole monotherapy (56)	21 (37.5)
Time to persistently afebrile, days	3 (1–6)
Concurrent other antibiotic use >3 days (56)	35 (62.5)
Time to persistently afebrile, days	2 (0–10)
Receipt of iodoquinol (60)	42 (70.0)
Operation and/or drainage (63)	14 (22.2)
Duration of follow-up (d) (57)	623.5 (10–3888)

Ag: antigen; ALP: alkaline phosphatase; GOT: aminotransferase; GPT: aminotransferase; HAART: highly active antiretroviral therapy; PCR: polymerase chain reaction; PVL: plasma HIV RNA load.

*: At diagnosis of invasive amebiasis.

### Sero-incidence of *E. histolytica* infection

**Figure 3:**
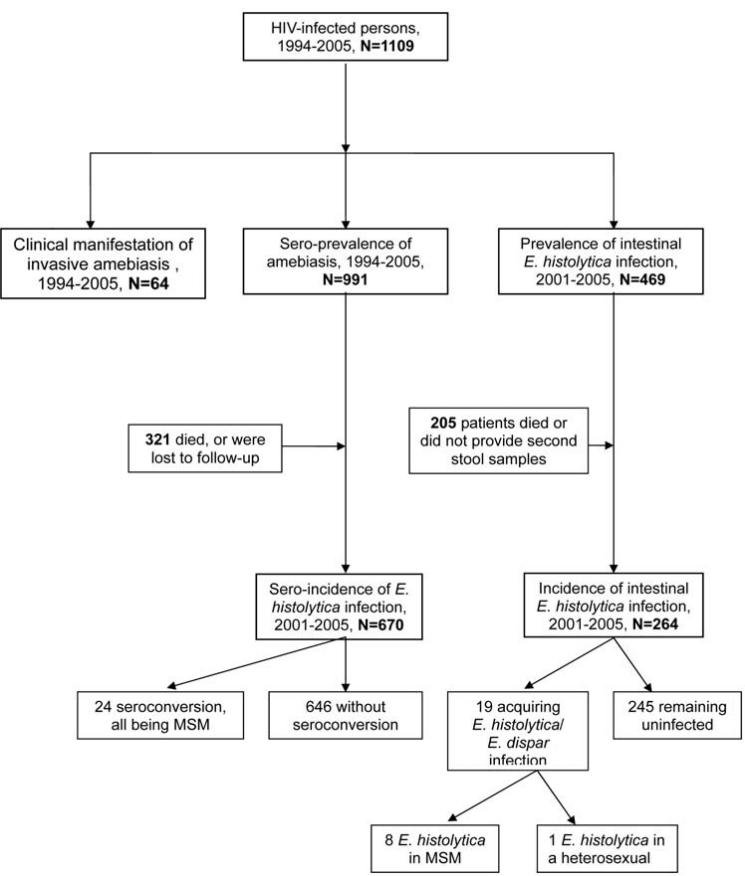
Study populations to determine the incidence of amebiasis among HIV-infected patients between 1994 and 2005.

Of 991 patients (89.4%) with available IHA assay results at baseline, 66 patients (6.7%) had IHA titers of 128 or greater. Between January 2001 and December 2005, 670 patients, including 433 (63.6%) MSM, who had more than one blood sample available for follow-up IHA assay, were enrolled in the sero-incidence study (Figure 3). There were no significant differences in demographics and clinical characteristics between the 670 patients who had follow-up IHA assays and the 321 who did not (data not shown). There were no significant differences in median CD4 count and plasma HIV RNA load (PVL) between MSM and patients from other risk groups when the first IHA assays were performed. At baseline, a significantly higher proportion of MSM (7.2%) had IHA titers of 128 or greater than patients from other risk groups (*p* = 0.006) (Table 2).

**Table 2:** Characteristics of non-hemophiliac patients aged 15 years or greater who had baseline and follow-up indirect hemagglutination (IHA) assays.

Variable	MSM	Heterosexuals and Other	All	Statistics, *p* =
Patients, N	433	237	670	
Sex, M/F	433/0	184/53	617/53	<0.0001
Age when first IHA was determined, median (IQR), y	32 (27, 38)	40 (32.5, 51)	35 (29, 42)	<0.0001
Age when second IHA was determined, median (IQR), y	36 (31, 42)	44 (38, 55)	39 (33, 46)	<0.0001
CD4 when first IHA was determined, median (IQR), cells/L	183 (45, 413)	135 (24, 323)	173 (37, 389)	0.02
CD4<200 cells/L, N (%)	195 (51.7)	102 (56.4)	297 (53.2)	0.30
CD4 when second IHA was determined, median (IQR), cells/L	400.5 (258, 593)	353 (223, 541)	385.5 (247.5, 574.5)	0.069
CD4<200 cells/L (%)	74 (18.4)	48 (21.6)	122 (19.6)	0.33
Interval between two IHA tests, median (IQR), days	958 (592,1700)	1184 (660, 2108)	1054 (606,1857)	0.002
HAART initiated, N (%)	401 (92.6)	228 (96.2)	629 (93.9)	0.07
Geometric mean IHA titer at baseline	134.19	48.11	100.83	0.16
IHA≧128, N (%)	31 (7.2)	5 (2.1)	36 (5.4)	0.006
Geometric mean IHA titer of the second test	108.89	33.90	89.64	0.0002
IHA≧128, N (%)	46 (10.6)	3 (1.3)	49 (7.3)	<0.0001
Total observation duration, PY	1458	963	2421	
*Seroconversion, N (%)	21 (4.9)	0 (0)	21 (3.1)	<0.0001
Incidence rate, per 100 PY (95% CI)	1.44 (0.89, 2.20)	0 (0, 0.38)	0.87 (0.54, 1.33)	<0.0001
**Seroconversion, N (%)	24 (5.5)	0 (0)	24 (3.6)	<0.0001
Incidence rate, per 100 PY (95% CI)	1.65 (1.05, 2.45)	0 (0, 0.38)	0.99 (0.64, 1.47)	<0.0001

Abbreviations: 95% CI, 95% confidence interval; 100 PY, 100 persons-years of observation; IDU: intravenous drug user; IQR, interquartile range; MSM, men who have sex with men.

Note: *Seroconversion from 0 at baseline to ≧128 at second tests. Rate ratio (MSM vs. patients of other risk groups): 13.87 (95% CI, 1.866, 103.1).

** Seroconversion from 0 at baseline to ≧128 at second tests; or from any titer at baseline to 4-fold rise or greater than baseline values. Rate ratio: 19.15 (95% CI, 2.61, 140.6).

The median interval between the two blood samples was 1054 days (interquartile range [IQR], 606–1857 days) (Table 2). Twenty-one (3.1%) of the 670 HIV-infected patients seroconverted with IHA titers from 0 to 128 or greater; the median interval for seroconvertion was 1507 days (IQR, 790–2039 days). MSM were at statistically significantly higher risk for seroconversion for *E. histolytica* infection. The crude sero-incidence of *E. histolytica* infection among MSM was 4.9% compared with 0% among other risk groups (*p*<0.0001). The incidence rate of seroconversion was 1.44 per 100 PY (95% CI, 0.89, 2.20 per 100 PY) among MSM, compared with 0 per 100 PY (95% CI, 0, 0.38 per 100 PY) among patients of other risk groups (*p*<0.0001). When an increase of IHA titer by 4-fold or greater was included along with changes of IHA titers from 0 to 128 or greater, the incidence rate of seroconversion was 1.99 per 100 PY (95% CI, 1.33, 2.86 per 100 PY) among MSM, compared with 0 (95% CI, 0, 0.38 per 100 PY) among patients of other risk groups (rate ratio, 19.15; 95% CI, 2.61, 140.6) (*p*<0.0001).

### Longitudinal survey of intestinal colonization with *E. histolytica*

Four hundred and sixty-nine (37.6%) patients, including 303 (64.6%) MSM, provided a total of 732 stool samples (range, 2–6 samples; median, 3 samples) for antigen testing between 2001 and 2005. At baseline, 45 (9.6%) patients, including 36 MSM (80%), had stool samples that tested positive for *E. histolytica*/*E. dispar* antigen. Two hundred and sixty-four patients, including 165 (62.5%) MSM, who had no intestinal infection with *E. histolytica*/*E. dispar* at baseline by specific stool antigen assays submitted more than one stool sample for repeat antigen assays (Table 3). At the first stool antigen assay, a significantly higher proportion of MSM (8.5%) had IHA titers of 128 or greater than patients from other risk groups (2.0%) (*p* = 0.03).

**Table 3:** Characteristics of non-hemophiliac patients aged15 years or greater who had baseline and follow-up stool amebic antigen assays.

Variable	MSM	Heterosexuals and Other	All	Statistics, *p* =
Patient number, N	165	99	264	
Sex, M/F	165/0	82/17	247/17	<0.0001
Age at first stool antigen assay, median (IQR), y	36 (31, 42)	44.5 (35, 52)	38 (32, 46)	<0.0001
Median stool samples submitted from each person, (range)	3 (2, 6)	3 (2, 6)	3 (2, 6)	0.69
CD4 when first stool antigen assay was performed, median (IQR), cells/L	295.5 (157, 490)	249 (129, 378)	282 (136, 445)	0.06
CD4<200, N (%)	50 (33.3)	38 (42.7)	88 (36.8)	0.17
CD4 at subsequent positive assays or the last negative assays, median (IQR), cells/L	372 (254, 541)	308 (185, 432)	336 (223, 512)	0.006
CD4<200 cells/L, N (%)	18 (13.0)	26 (31.3)	44 (19.8)	0.002
Interval between the first negative stool antigen and subsequent positive assays or the last negative assays, median (IQR), days	423 (244, 933)	451 (258, 771)	430.5 (245, 826.5)	0.83
HAART initiated, N (%)	152 (92.1)	98 (99.0)	250 (94.7)	0.02
Geometric mean IHA titers at baseline	322.54	90.51	256	0.045
IHA≧128, N (%)	14 (8.48)	2(2.02)	16(6.06)	0.03
Total observation duration, PY	252	147	399	
From first antigen-negative at baseline to first antigen-positive tests, N (%)	14 (8.5)	5 (5.1)	19 (7.2)	0.37
Interval between two tests, median (IQR), d	279.5 (221, 393)	317 (245, 319)	287 (221, 393)	0.93
Incidence rate of *E. histolytica*/ *E. dispar* infection, per 100 PY (95% CI)	5.56 (3.04, 9..32)	3.40 (1.10, 7.94)	4.76 (2.87, 7.43)	0.36
From first antigen-negative at baseline to the first antigen-positive and PCR-positive tests, N (%)	8 (4.85)	1 (1.01)	9 (3.41)	0.16
Interval between two tests, median (IQR)	459 (311, 747.5)	245	427 (245, 708)	0.44
*Incidence rate of *E. histolytica* infection, per 100 PY (95% CI)	3.16 (1.37, 6.23)	0.68 (0.02, 3.79)	2.26 (1.03,4.28)	0.12

Abbreviations: 95% CI, 95% confidence interval; 100 PY, 100 persons-years of observation;

IDU: intravenous drug user; IHA: indirect hemagglutination; IQR, interquartile range; MSM, men who have sex with men; PCR, polymerase chain reaction.

Note: *Rate ratio (MSM vs. patients of other risk groups): 4.083 (95% CI, 0.5025, 33.18).

After an observation period of 399 PY, 19 (7.2%) patients were found to be infected with *E. histolytica*/*E. dispar*; 9 (47.4%) of the 19 isolates were *E. histolytica* by PCR. The median interval between the negative and positive antigen tests of the 19 patients was 287 days (IQR, 221–393 days). The crude incidence of new *E. histolytica*/*E. dispar* infection among MSM was 8.5%, compared with 5.1% among patients from other risk groups (*p* = 0.37); the incidence rate of new acquisition of *E. histolytica*/*E. dispar* infection was 5.56 per 100 PY (95% CI, 3.04, 9..32 per 100 PY) among MSM, compared with 3.40 per 100 PY (95% CI, 1.10, 7.94 per 100 PY) among patients from other risk groups (*p* = 0.36). Of the 9 *E. histolytica* isolates, 8 isolates were from 165 MSM, while 1 was from 99 patients from other risk groups (*p* = 0.16). The incidence rate of *E. histolytica* infection was 3.16 per 100 PY (95% CI, 1.37, 6.23 per 100 PY) among MSM compared with 0.68 per 100 PY (95% CI, 0.02, 3.79 per 100 PY) among patients from other risk groups with a rate ratio of 4.667 (95% CI, 0.5837, 37.31) (*p* = 0.12). All of the patients with *E. histolytica* infection were asymptomatic.

Six of 9 (66.7%) patients, who acquired new *E. histolytica* infection, seroconverted from sero-negative to sero-positive for anti-*E. histolytica* antibodies, compared with only 2 of 255 (0.78%) who did not acquire *E. histolytica* infection (odds ratio, 251 [95% CI, 35.22, 1789]) (*p*<0.0001).

**Figure 4:**
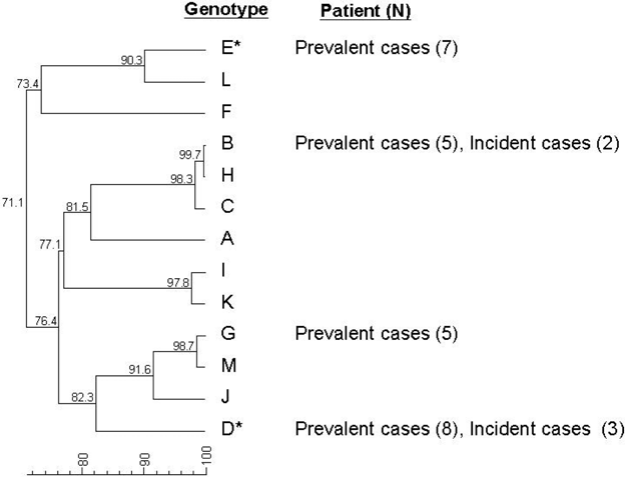
Results *E. histolytica* genotyping.

The results from sequencing extracted DNA from the *E. histolytica* isolates in this study and other isolates of *E. histolytica* from our previous prevalence study [31] are shown in Figure 4. Case clustering among isolates from MSM was noted (*Locus*1-2 allelic genotypes B and D), suggesting a common source or person-to-person transmission. However, geographical unrelatedness among those patients with intestinal *E. histolytca* infection suggests that person-to-person transmission of *E. histolytica* might have occurred among MSM.

In univariate analysis, patients acquiring amebiasis were predominantly MSM and had significantly higher CD4 counts than those who remained uninfected (315 vs. 157 cells/L; *p*<0.001) (data not shown). In multiple logistic regression analysis, we found that MSM was the only risk factor that was associated with new acquisition of *E. histolytica* infection by serologies or antigen assays followed by PCR, with an adjusted odds ratio of 14.809 (95% CI, 1.824, 120.237; *p* = 0.01) when compared with heterosexuals or patients with other risk behaviors (data not shown). The adjusted odds ratio for new acquisition of *E. histolytica* infection for every 50-cells/L CD4 increase or 1−log_10_ copies/ml plasma HIV RNA load decrease was 1.066 (95% CI, 0.975, 1.167; *p* = 0.16) and 1.180 (95% CI, 0.813, 1.713; *p* = 0.38), respectively.

## Discussion

This is the first longitudinal follow-up study to investigate the incidence of *E histolytica* infection in HIV-infected patients by examining the incidence rate of intestinal *E. histolytica* infection and seroconversion of anti-*E. histolytica* antibodies. We found that HIV-infected MSM were at significantly higher risk for acquisition of *E. histolytica* infection [31],[34]. Despite immunosuppression from HIV infection and the complicated disease course of IA, clinical responses to metronidazole therapy were favorable in terms of rapid defervescence and a low attributable mortality rate.

Exposure to *E. histolytica*, but not *E. dispar*, may induce anti-*E. histolytica* antibody response and development of anti-*E. histolytica* antibodies may represent either recent or remote exposure to *E. histolyica* [14],[42], although not every person infected with *E. histolytica* develops an antibody response. Our analysis also showed that acquisition of *E. histolytica* infection was significantly associated with seroconversion for anti-*E. histolytica* antibodies despite immunosuppression from HIV infection. Therefore, such a test may be used as a complimentary tool to understand the epidemiology of *E. histolytica* among high-risk populations. By using serological surveys, Japanese investigators have found that the seroprevalence of *E. histolytica* infection in MSM was as high as 13.4–20.4% compared with 1.0% in heterosexuals and 0.8% in prostitutes [24]–[26],[33]. Similarly, we found that the seroprevalence of *E. histolytica* infection among HIV-infected patients remained significantly higher compared with HIV-uninfected persons with gastrointestinal symptoms who had their sera tested for anti-*E. histolytica* antibodies [34].

In this study, we further explored the sero-incidence of *E. histolytica* infection in HIV-infected patients. The results also indicate that MSM are at increased risk of exposure to *E. histolytica* infection. The findings of higher seroprevalence and sero-incidence of MSM, regardless of HIV status, are caused by higher prevalence and incidence of intestinal infection with *E. histolytica*. In our previous study, we found that the prevalence of *E. histolytica*/*E. dispar* by stool antigen tests was 12.1%, compared with 1.4% healthy controls; and at least 25% of the isolates from HIV-infected persons were confirmed as *E. histolytica* by PCR [31]. Although the majority of persons infected with *E. histolytica* are asymptomatic [1],[2], more than 80% of the 600 or greater annually reported cases of amebiasis in Japan occurred in MSM [32]. These findings may reflect a decrease in *E. histolytica* infection in developed countries where improvement of public hygiene and sanitation has reduced the risk of acquisition of *E. histolytica* through contaminated water or food.

Sharing the identical transmission route with *E. histolytica*, the transmission of *E. dispar* among MSM in developed countries is correlated with oral-anal sexual contact and 20–40% of MSM who visited the sexually transmitted diseases clinic were found to be infected with *E. dispar* [10]–[12]. Therefore, infection with either *E. dispar* or *E. histolytica* is indicative of unsafe oral-anal sexual contact among MSM. In this study, we further demonstrated that HIV-infected MSM were more likely than other risk groups to acquire *E. histolytica* infection during follow-up, although this finding is not statistically significantly different due to the small sample size. Nearly 4% of HIV-infected MSM acquired *E. histolytica*, compared with 1% among patients from other risk groups. Furthermore, case clustering that was identified by molecular typing of the isolates occurred probably through person-to-person transmission. These findings highlight the importance of counseling MSM about precautions to prevent acquiring *E. histolytica* infection through oral-anal sexual contact.

The clinical manifestations of IA in our HIV-infected patients with significant immunosuppression were similar to those previously described in HIV seronegative patients. Amebic colitis and liver abscesses were the two most common presentations [1],[2],[29]. The severity of the diseases was reflected by the high proportion of liver abscesses (59.7%) and complications (11.9%) in our cases. Despite low CD4 counts upon diagnosis with IA, the responses to metronidazole therapy with or without combination with antibiotics were favorable, as shown by rapid defervescence within 2 days of therapy initiation and no death attributable to IA.

There are several limitations of our study. First, the risk for exposure to *E. histolytica* is low in the general population in Taiwan, as reflected by the low seroprevalence (0.12%) of *E. histolytica* infection among 2500 healthy controls in a recent survey in northern and southern Taiwan [34]. Therefore, generalizations about our findings to areas of higher endemicity of *E. histolytica* and HIV infection should be cautious. Second, most patients at the late stage of HIV infection who develop HIV-related complications are referred to this hospital. However, those patients with IA had significantly higher CD4 counts than the patients without IA, suggesting that *E. histolyica* infection may not be associated with immunosuppression in HIV-infected patients. Rather, it is the risky behavior that increases risk of *E. histolyica* infection and subsequent development of invasive diseases. Third, our study was limited by the small sample size in assessment of the incidence of *E. histolytica* infection by stool antigen assays during follow-up. Although the incidence of *E. histolytica* infection is higher in MSM than in heterosexuals and others, the difference does not reach statistical significance. The shedding of *E. histolytica* may be intermittent, which may reduce the sensitivity of antigen assays if only one stool sample is tested. However, combinations with IHA assays for *E. histolytica* infection in our study may compensate for this deficiency by increasing the detection sensitivity. In this study, we chose a high titer of 128 as the cut-off value which decreases the possibility of cross-reactions, and seroconversion was significantly associated with newly acquired *E. hisitolytica* infection. Last, our genotyping methods [40],[41] may not be as sensitive enough for detection of genetic differences between the isolates as the new genotyping system that uses 6 tRNA-linked short tandem repeats by Ali and colleagues [43].

In conclusion, HIV-infected MSM in Taiwan are at a higher risk of acquisition of *E. histolytica* infection and IA than other HIV-infected patients. It should also be investigated whether this is the case in other countries. Certainly physicians, treating MSM with or without HIV infection, should be aware of this potential complication, that until recently, in industrialized countries was seen nearly only in travelers returning from *E. histolytica* endemic regions.

## References

[1] Haque R, Huston CD, Hughes M, Houpt E, Petri WA (2003). Amebiasis.. N Engl J Med.

[2] Stanley SL (2003). Amoebiasis.. Lancet.

[3] Lucas SB (1990). Missing infections in AIDS.. Trans R Soc Trop Med Hyg.

[4] Reed SL, Wessel DW, Davis CE (1991). *Entamoeba histolytica* infection and AIDS.. Am J Med.

[5] Smith PD, Lane HC, Gill VJ, Manischewitz JF, Quinnan GV (1988). Intestinal infections in patients with the acquired immunodeficiency syndrome (AIDS). Etiology and response to therapy.. Ann Intern Med.

[6] Blanshard C, Collins C, Francis N, Gazzard BG (1992). Invasive amoebic colitis in AIDS patients.. AIDS.

[7] Fatkenheuer G, Arnold G, Steffen HM, Franzen C, Schrappe M (1997). Invasive amoebiasis in two patients with AIDS and cytomegalovirus colitis.. J Clin Microbiol.

[8] Weber R, Ledergerber B, Zbinden R, Altwegg M, Pfyffer GE (1999). Enteric infections and diarrhea in human immunodeficiency virus-infected persons: prospective community-based cohort study. Swiss HIV Cohort Study.. Arch Intern Med.

[9] Lowther SA, Dworkin MS, Hanson DL, and the Adult and Adolescent Spectrum of Human Immunodeficiency Virus Disease Project (2000). *Entamoeba histolytica*/*Entamoeba dispar* in human immunodeficiency virus-infected patients in the United States.. Clin Infect Dis.

[10] Phillips SC, Mildvan D, William DC, Gelb AM, White AC (1981). Sexual transmission of enteric protozoa and helminths in a venereal-disease-clinic population.. N Engl J Med.

[11] Quinn TC, Stamm WE, Goodell SE, Mkrtichian E, Benedetti J (1983). The polymicrobial origin of intestinal infections in homosexual men.. N Engl J Med.

[12] Allason-Jones E, Mindel A, Sargenunt P, Williams P (1986). *Entamoeba histolytica* as a commensal intestinal parasite in homosexual men.. N Engl J Med.

[13] Sorvillo FJ, Strassburg MA, Seidel J, Visvesvara GS, Mori K (1986). Amebic infections in asymptomatic homosexual men, lack of evidence of invasive disease.. Am J Public Health.

[14] Tanyuksel M, Petri WA (2003). Laboratory diagnosis of amebiasis.. Clin Microbiol Rev.

[15] Gomez Morales MA, Atzori C, Ludovisi A, Rossi P, Scaglia M (1995). Opportunistic and non-opportunistic parasites in HIV-positive and negative patients with diarrhoea in Tanzania.. Trop Med Parasitol.

[16] Germani Y, Minssart P, Vohito M, Yassibabda S, Glaziou P (1998). Etiologies of acute, persistent, and dysenteric diarrheas in adults in Bangui, Central African Republic, in relation to human immunodeficiency virus serostatus.. Am J Trop Med Hyg.

[17] Fontanet AL, Sahlu T, Rinke de Wit T, Messele T, Masho W (2000). Epidemiology of infections with intestinal parasites and human immunodeficiency virus (HIV) among sugar-estate residents in Ethiopia.. Ann Trop Med Parasitol.

[18] Waywa D, Kongkriengdaj S, Chaidatch S, Tiengrim S, Kowadisaiburana B (2001). Protozoan enteric infection in AIDS related diarrhea in Thailand.. Southeast Asian J Trop Med Public Health.

[19] Mohandas K, Sehgal R, Sud A, Malla N (2002). Prevalence of intestinal parasitic pathogens in HIV-seropositive individuals in Northern India.. Jpn J Infect Dis.

[20] Senya C, Mehta A, Harwell JI, Pugatch D, Flanigan T (2003). Spectrum of opportunistic infections in hospitalized HIV-infected patients in Phnom Penh, Cambodia.. Int J STD AIDS.

[21] Hailemariam G, Kassu A, Abebe G, Abate E, Damte D (2004). Intestinal parasitic infections in HIV/AIDS and HIV seronegative individuals in a teaching hospital, Ethiopia.. Jpn J Infect Dis.

[22] Sadraei J, Rizvi MA, Baveja UK (2005). Diarrhea, CD4+ cell counts and opportunistic protozoa in Indian HIV-infected patients.. Parasitol Res.

[23] Moran P, Ramos F, Ramiro M, Curiel O, González E (2005). Infection by human immunodeficiency virus-1 is not a risk factor for amebiasis.. Am J Trop Med Hyg.

[24] Takeuchi T, Kobayashi S, Asami K, Yamaguchi N (1987). Correlation of positive syphilis serology with invasive amebiasis in Japan.. Am J Trop Med Hyg.

[25] Takeuchi T, Miyahira Y, Kobyash S, Nozaki T, Motta S (1980). High seropositivity for *Entamoeba histolytica* infection in Japanese homosexual men: further evidence for occurrence of pathogenic strains.. Trans R Soc Trop Med Hyg.

[26] Takeuchi T, Okuzawa E, Nozaki T, Kobayashi S, Mizokami M (1989). High seropositivity of Japanese homosexual men for amebic infection.. J Infect Dis.

[27] Ohnishi K, Murata M (1997). Present characteristics of symptomatic amebiasis due to *Entamoeba histolytica* in the east-southeast area of Tokyo.. Epidemiol Infect.

[28] Hung CC, Chen PJ, Hsieh SM, Wong JM, Fang CT (1999). Invasive amebiasis: an emerging parasitic disease in patients with HIV infection in an endemic area of amebic infection.. AIDS.

[29] Park WB, Choe PG, Jo JH, Kim SH, Bang JH (2007). Amebic liver abscess in HIV-infected patients, Republic of Korea.. Emerg Infect Dis.

[30] Ohnishi K, Kato Y, Imamura A, Fukayama M, Tsunoda T (2004). Present characteristics of symptomatic *Entamoeba histolytica* infection in the big cities of Japan.. Epidemiol Infect.

[31] Hung CC, Deng HI, Hsiao WH, Hsieh SM, Hsiao CF (2005). Invasive amoebiasis is an emerging parasitic infection in patients with HIV infection.. Arch Intern Med.

[32] Nozaki T, Kobayashi S, Takeuchi T, Haghighi A (2006). Diversity of clinical isolates of *Entamoeba histolytica* in Japan.. Arch Med Res.

[33] Aceti A, Pennica A, Ippolito G (1987). Antiamebic antibodies in homosexual men.. N Engl J Med.

[34] Tsai JJ, Sun HY, Ke LY, Tsai KS, Chang SY (2006). Higher seroprevalence of *Entamoeba histolytica* infection is associated with human immunodeficiency virus Type 1 infection in Taiwan.. Am J Trop Med Hyg.

[35] Ko NY, Lee HC, Chang JL, Lee HY, Chang CM (2006). Prevalence of human immunodeficiency virus and sexually transmitted infections and risky sexual behaviors among men visiting gay bathhouses in Taiwan.. Sex Transmitted Dis.

[36] Stark DJ, Fotedar R, Ellis JT, Harkness JL (2006). Locally acquired infection with *Entamoeba histolytica* in men who have sex with men in Australia.. Med J Aust.

[37] Keystone JS, Keystone DL, Proctor EM (1980). Intestinal parasitic infections in homosexual men: prevalence, symptoms and factors in transmission.. CMAJ.

[38] Liu CJ, Hung CC, Chen MY, Lai YP, Chen PJ (2001). Amebic liver abscess and human immunodeficiency virus infection: a report of three cases.. J Clin Gastroenterol.

[39] Centers for Disease Control and Prevention (1992). 1993 revised classification system for HIV infection and expanded surveillance case definition for AIDS among adolescents and adults.. MMWR Recomm Rep.

[40] Haghighi A, Kobayashi S, Takeuchi T, Masuda G, Nozaki T (2002). Remarkable genetic polymorphism among *Entamoeba histolytica* isolates from a limited geographic area.. J Clin Microbiol.

[41] Zaki M, Clark CG (2001). Isolation and characterization of polymorphic DNA from *Entamoeba histolytica*.. J Clin Microbiol.

[42] Gathiram V, Jackson TFHG (1987). A longitudinal study of asymptomatic carriers of pathogenic zymodemes of *Entamoeba histolytica*.. S Afr Med J.

[43] Ali IKM, Zaki M, Clark CG (2005). Use of PCR amplification of tRNA gene-linked short tandem repeats for genotyping *Entamoeba histolytica*.. J Clin Microbiol.

